# CAN Canopy Addition of Nitrogen Better Illustrate the Effect of Atmospheric Nitrogen Deposition on Forest Ecosystem?

**DOI:** 10.1038/srep11245

**Published:** 2015-06-10

**Authors:** Wei Zhang, Weijun Shen, Shidan Zhu, Shiqiang Wan, Yiqi Luo, Junhua Yan, Keya Wang, Lei Liu, Huitang Dai, Peixue Li, Keyuan Dai, Weixin Zhang, Zhanfeng Liu, Faming Wang, Yuanwen Kuang, Zhian Li, Yongbiao Lin, Xingquan Rao, Jiong Li, Bi Zou, Xian Cai, Jiangming Mo, Ping Zhao, Qing Ye, Jianguo Huang, Shenglei Fu

**Affiliations:** 1 Key Laboratory of Vegetation Restoration and Management of Degraded Ecosystems, South China Botanical Garden, Chinese Academy of Sciences, Guangzhou 510650, China; 2 State Key Laboratory of Cotton Biology, Key Laboratory of Plant Stress Biology, College of Life Sciences, Henan University, Kaifeng, Henan 475004, China; 3 Department of Microbiology and Plant Biology, University of Oklahoma, Norman, OK 73019, USA; 4 State Key Laboratory of Urban and Regional Ecology, Research Center for Eco-Environmental Sciences, Chinese Academy of Sciences, Beijing 100085, China; 5 Jigongshan National Natural Reserve, Xinyang, Henan 464000, China; 6 Shimentai National Natural Reserve, Yingde, Guangdong 513000, China

## Abstract

Increasing atmospheric nitrogen (N) deposition could profoundly impact community structure and ecosystem functions in forests. However, conventional experiments with understory addition of N (UAN) largely neglect canopy-associated biota and processes and therefore may not realistically simulate atmospheric N deposition to generate reliable impacts on forest ecosystems. Here we, for the first time, designed a novel experiment with canopy addition of N (CAN) vs. UAN and reviewed the merits and pitfalls of the two approaches. The following hypotheses will be tested: i) UAN overestimates the N addition effects on understory and soil processes but underestimates those on canopy-associated biota and processes, ii) with low-level N addition, CAN favors canopy tree species and canopy-dwelling biota and promotes the detritus food web, and iii) with high-level N addition, CAN suppresses canopy tree species and other biota and favors rhizosphere food web. As a long-term comprehensive program, this experiment will provide opportunities for multidisciplinary collaborations, including biogeochemistry, microbiology, zoology, and plant science to examine forest ecosystem responses to atmospheric N deposition.

There is a growing consensus that human activities have induced dramatic and unprecedented changes in global chemical and physical environment over the 20^th^ century^1^. As a result of an increase in fossil fuel combustion, production and application of N fertilizer, the reactive N on the Earth has increased dramatically since the industry revolution, far exceeding the N demand of various ecosystems^2^. It is estimated that the global rate of N deposition has increased from approximately 34 Tg N yr^−1^ in 1860 to 100 Tg N yr^−1^ in 1995, and will continue to increase up to 200 Tg N yr^−1^ by 2050^3^. In recent decades, high rates of atmospheric N deposition have been widely documented in Europe, North America, and East Asia^4^,^5^,^6^. China, as a rapidly developing country, is experiencing intensifying N deposition in its central and southeastern areas^7^. Moreover, the trend of N deposition over China is predicted to dramatically increase in the coming decades^8^.

Reactive N deposited from the atmosphere to the land and water body can cause cascading environmental problems, including environmental pollution, biodiversity loss, greenhouse gas emissions, soil acidification, and degradation of terrestrial and aquatic ecosystems^9^,^10^,^11^,^12^,^13^,^14^. As one of the global change drivers, increasing atmospheric N deposition has the potential to shift the structure and functions of forest ecosystems^3^,^9^,^15^. What are the long-term consequences of N deposition in terms of forest structure and functions? What is the ultimate fate of the deposited N? How to better simulate the natural processes of atmospheric N deposition using manipulative experiments in the field? Great research efforts have been devoted to address these questions over the last two decades. Our current knowledge of N deposition impacts on forest ecosystems largely derives from numerous field manipulative experiments^12^,^13^,^16^,^17^,^18^,^19^. However, almost all the previous experiments mimicking N deposition have been conducted by adding N solution and/or fertilizer directly onto the understory plants and/or forest floors. Given that the conventional experiments with UAN neglect many components and processes occurring in canopy that are critical for forest ecosystems, an innovative technology that can better simulating atmospheric N deposition is urgently needed to improve mechanistic understanding, model simulation and projection on forests in response to N enrichment.

This paper described a novel aspect of N deposition manipulation, CAN vs. UAN, to examine forest ecosystem responses to atmospheric N deposition. The aims of this study were: i) to provide a comprehensive analysis of the past and ongoing manipulative experiments of N deposition, ii) to introduce a novel approach-CAN, which can more realistically simulate and quantify the forest responses to increasing N deposition, and iii) to test whether the traditional approach-UAN have overestimated the effects of N addition on understory species and soil processes but underestimated the effects on canopy processes.

## Current studies of N deposition on forest ecosystems

Concerns about the potential impacts of N deposition on forest ecosystems have led to increasing studies over the last two decades^4^,^12^,^13^,^16^,^18^,^19^,^20^,^21^. As a common technology, different forms of N fertilizer including urea, NH_4_NO_3_, NaNO_3_, (NH_4_)_2_SO_4_, and NH_4_Cl are directly applied to the forest floor, or indirectly by dissolving into water and spraying the N solutions onto the understory plants and/or forest floors with target rates (e.g., the same or double of ambient N deposition rate). These experiments have provided valuable assessments on the responses of forests to elevated N deposition. In N-limited or young forests, enhanced N availability can stimulate plant growth and cause a substantial CO_2_ sequestration^20^,^22^. By contrast, excessive N deposition can impose negative impacts on forest structure and functions, including loss of biodiversity^10^,^14^, invasion of exotic species^23^, soil acidification^12^,^19^, eutrophication and N saturation^11^,^16^,^24^, and declines in forest growth^25^. Based the conventional N manipulative experiments, three major consensus have been reached: i) the responses of forest ecosystems to N enrichment vary with the duration of N additions, forest history, and soil characteristics, ii) low N deposition facilitates plant growth and gross primary productivity (GPP), but excess N input reduces forest productivity, and iii) a tipping point (threshold load) may exist where forest ecosystems do not respond to extra N addition, and this status refers to “N saturation”^24^.

Nevertheless, the past and ongoing experimental designs can rarely mimic the realistic way by which atmospheric N is naturally deposited to forest ecosystems, posing challenges for mechanistic understanding, convincingly simulating and projecting the patterns and dynamics of forest ecosystems in the future. It is well known that deposited N passes through the canopy layer before it reaches forest floor^26^. When N is directly deposited to canopy, it becomes immediately available for uptake by plant leaves and subsequently promotes photosynthesis and leads to an increase in production^27^. The composition of inorganic N can be changed during passage through the canopy with variations of NH_4_^+^:NO_3_^−^ ratios^28^. However, previous N manipulative experiments with the UAN-approach did not include the effects of atmospheric N deposition on the canopy-associated biota and processes.

Nitrogen retention by the forest canopy is an important process of biogeochemical cycles^29^,^30^, and its quantification is a key issue in determining the impacts of atmospheric N deposition on forest ecosystems. What happens to the N that is retained in the canopy? Potential fates include: uptake by canopy tree leaves, epiphytes and microorganisms^31^; chemical retention in or on the barks^32^; immobilization in decaying leaves, twigs or other dead organic matters in the canopy^33^; volatilization as water evaporates^34^; and transformation of inorganic N to organic N^35^. There are strong evidences showing that canopy N retention depends on forest type, ambient N deposition amount, and soil characteristics. Adriaenssens *et al.* (2012) reported that only 1–5% of the applied dissolved ^15^N was taken up by leaves and twigs, as a minor fraction of high ambient N deposition (north Belgium, 30 kg N ha^−1^yr^−1^)^36^. Wortman *et al.* (2012) found that the canopy retained 20–25% of the total atmospheric deposited N at the Novaggio forest, Switzerland (experiencing high rates of N deposition as 25–40 kg N ha^−1^yr^−1^)^27^. In a low ambient N deposition area (<5 kg N ha^−1^yr^−1^), Gaige *et al.* (2007) demonstrated a 70% retention of added N in the canopy at the Howland Integrated Forest Study site (east-central Maine, USA)^37^, but a later study reported that 10–25% of the ^15^N was retained in or on twig and branch materials whereas only 3–6% was recovered in live foliage and bole wood^32^. The poor recovery of ^15^N tracer was explained by volatilized, unmeasured or incompletely measured fluxes such as denitrification, leaching, and photolysis which could cause NO_3_^-^ loss^32^,^38^. Moreover, at the Niwot Ridge AmeriFlux site (Colorado, USA) in a low ambient N deposition area (3 kg N ha^−1^yr^−1^), approximately 80% of the growing season total N deposition was retained in canopy foliage and branches^39^. If the retained N in canopy is fully utilized and immobilized by trees or epiphytes, most of them can eventually enter the soil via litterfall decomposition^40^,^41^. However, plants, but not soils, are the short-term sink for canopy added N. Therefore, the responses of canopy-associated biota and processes have been largely ignored in those manipulative experiments with UAN-technology.

There have been very few researches implemented using new technology with an attempt to better simulate the natural N deposition processes in forest canopies. Using a helicopter to spray a fine mist of liquid NH_4_NO_3_ onto the canopy of a 21-ha area on five different dates during the growing season (from May to August) in low-elevation transition spruce-fir forests in east-central Maine, USA, Gaige *et al.* (2007) and Dail *et al.* (2009) examined the response of a mature coniferous forest to N deposition^32^,^37^, but this program was terminated after four years possibly due to expensive cost. Another excellent whole-watershed study on evaluating acidic deposition effects on temperate forests, with three aerial applications of (NH_4_)_2_SO_4_ annually by a low flying fixed-wing aircraft, has been carrying out in the Fernow Experimental Forest (FEF), West Virginia, USA since 1989^11^,^15^,^21^,^25^,^42^,^43^,^44^,^45^,^46^. A paired experiment using the same approach has also been conducting in a temperate forest in the Bear Brook Watershed of Maine, USA since 1989^47^. Sheppard *et al.* (1999) and Cape *et al.* (2010) also investigated the response of a Sitka spruce (*Picea sitchensis* (Bong.) Carr) plantation in south-central Scotland to N deposition by using a canopy spraying system to spray N solution onto the canopy^48^,^49^. Nevertheless, neither of these research programs has tried to compare the possibly different impacts of canopy and understory N addition on forest trees and ecosystems.

## Introduction to the CAN vs. UAN facilities

### Experimental sites

**Figure 1:**
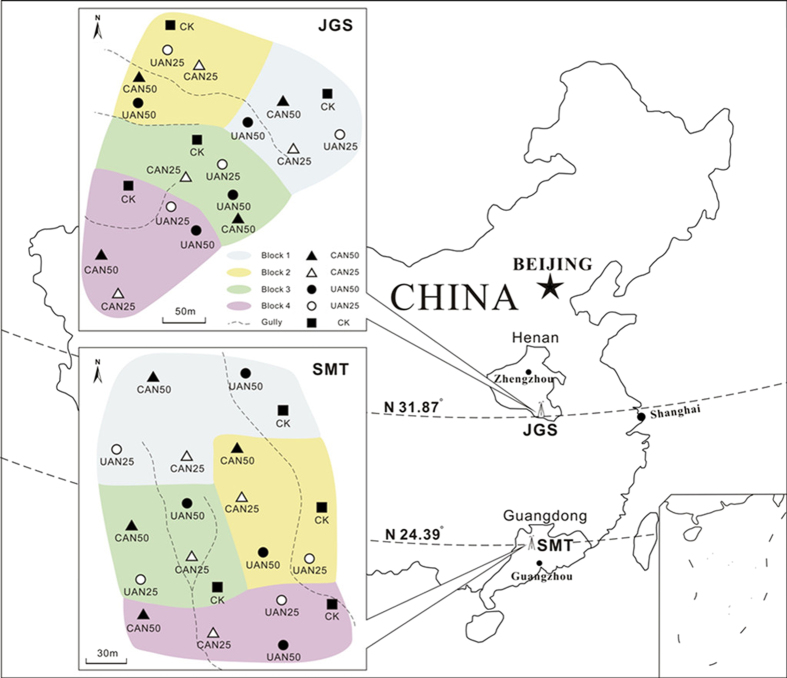
Location of experimental sites and layout of treatment plots at each site. JGS, Jigongshan National Natural Reserve, located in Henan Province of China; SMT, Shimentai National Natural Reserve, located in Guangdong Province of China. The original map was from Data Sharing Infrastructure of Earth System Science of China. This map was created using ESRI ArcGis10.0 software. Figure drawing by J. Li.

The N manipulative experiments are conducted in Central and South China, respectively (Fig. 1). The impacts of the two different N manipulation approaches, CAN vs. UAN, on forest structure and functions are being investigated at both sites. The Jigongshan (JGS) forest site is located in the Jigongshan National Nature Reserve (31°46'-31°52' N, 114°01'-114°06' E) of Henan Province, Central China, within a transitional zone from subtropical climate to warm temperate climate region. The Shimentai (SMT) forest site is located in the Shimentai National Nature Reserve (24°22'-24°31' N, 113°05'-113°31' E) of Guangdong Province, South China. The region is dominated by a subtropical monsoon climate with alternating moist and dry seasons. Site information and community structure characteristics of both sites are given in Table 1.

### Experimental treatment

The CAN vs. UAN experiment is a full factorial design for elevated N application, with five different levels of treatments (Fig. 1). The treatments include: i) canopy addition of N 25 kg N ha^−1^yr^−1^ (CAN25); ii) canopy addition of N 50 kg N ha^−1^yr^−1^ (CAN50); iii) understory addition of N 25 kg N ha^−1^yr^−1^ (UAN25); iv) understory addition of N 50 kg N ha^−1^yr^−1^ (UAN50); and v) control (C, without N addition). Four blocks were established at each forest site, and each treatment was replicated once within each of the four blocks. Within each block, the five treated plots were randomly assigned. For each site, a total of 20 circular plots were established corresponding to five treatments with four replicates. The semi-diameter for each circular plot is 17 m with an area of 907 m^2^, leaving the central core area of 400 m^2^ for sampling of plant and soil variables and other area for side projects. Lateral contamination of N solution between plots was minimal as the plots were separated by at least 20 m buffer zone, and polyvinylchloride (PVC) boards were inserted between two adjacent plots when necessary.

The treatments for the CAN and UAN were initiated in April 2013, all the background data were collected before the first treatment. This program is designed to run for at least ten years and samples of plant, soil, and water will be collected periodically. Major parameters to be monitored include, but not limited to, plant performance (survival and growth), functional traits (penology, photosynthesis, water relations, nutrients, etc.), epiphytes (orchids, lichins and algae), litterfall (production and decomposition), fine root dynamics (production, mortality, and turnover), soil biotic activity (soil microorganisms, animals and enzymes), plant species diversity, C and N fluxes, and soil C stability. In the coming years, atmospheric N deposition will be continuously monitored at both sites. The dry deposition of N components were collected by the DELTA (DEnuder for Long-term Atmospheric Sampling) system, which is an active sampling equipment, fulfilling long-time sampling and thus cost saving^50^. Wet deposition of N was collected *in situ* by precipitation collectors (SDM6, Tianjin Weather Equipment Inc., China). The detailed descriptions on samples analysis and N deposition calculation can be found by Luo *et al.* (2014)^51^. Meteorological variables such as wind direction, wind speed, solar radiation, rainfall, air temperature, and soil temperature (0, 10 and 20 cm) at each study site were recorded as 15- or 30-min averages, using an Automatic Meteorological Station (FM1000, Campbell Co., England).

### Main components and operation of CAN and UAN

**Figure 2:**
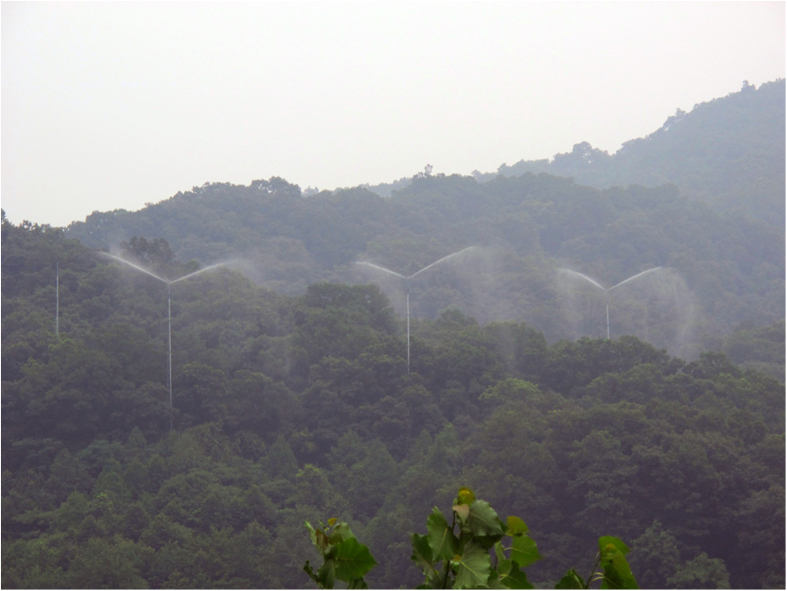
CAN facility at JGS site showing three spraying systems in normal treatment. The photograph was taken at Jigongshan Forest. The regional vegetation at JGS is subtropical to temperate transient deciduous forest. Photograph credit: J. Li.

A forest canopy spraying system was built, in the center of plot where canopy N addition was needed, to deliver the N solutions. The system consisted of five components: a tank for N solution storage, connecting pipes, a supporting tower, four sprinklers and a central computer controller. A tank with the capacity of 20 m^3^ were built to store N solution at the upside of the experimental site and connected with different PVC pipes (7.5 cm inner diameter) to transport N solution to the top of the supporting tower. The supporting tower is made of galvanized steel, standing 35 m high with a strong concrete base (200 cm × 200 cm × 200 cm), to support the PVC pipes and four crane sprinklers. To quantify the actual amount of the solution used, flow meters and pressure gauges are installed at the ends of the pipes. Four sprinklers with different spraying range are installed on the tower top, which are 5 m above the canopy. The sprinklers can turn 360 degree and spray the solution as far as 17 m depending upon the given pressure (Fig. 2). Solutions were conveyed to the sprinklers by high pressure water pumps. All the technical parameters were set and controlled by a central computer. Nitrogen solution of targeted concentration was made on site by weighing the appropriate amount of NH_4_NO_3_ salt and mixing it into the surface water drained from an upper-position lake and sprayed onto the canopy. To ensure each spraying N concentration being the target concentration, lake water is sampled and analyzed before each N application event. The consumption of solution was at a rate of 3 mm precipitation equivalent per application event, such treatments were performed monthly from April to October (7 times in a year). The total solution used is 21 mm per year, accounting for <1% and <2% of total annual precipitation of SMT and JGS, respectively. Therefore, the confounding effect caused by water addition is negligible in this study. The pattern and frequency of spraying were determined by the phenological of JGS forest. Normally, the first treatment was started roughly a week before all buds had flushed (around 15 April), and the final treatment was achieved in the middle of October when litter started to fall. Spraying events were carried out in the morning or evening on days when wind speed was <1 m/s with minimal sunshine. Nitrogen solutions for UAN were sprinkled 1.5 m above from the ground by means of an automatic irrigation system, which made up of 5 sprinklers and evenly distributed in each plot. The frequency and duration of UAN were the same as the application event of CAN at each study site.

**Figure 3:**
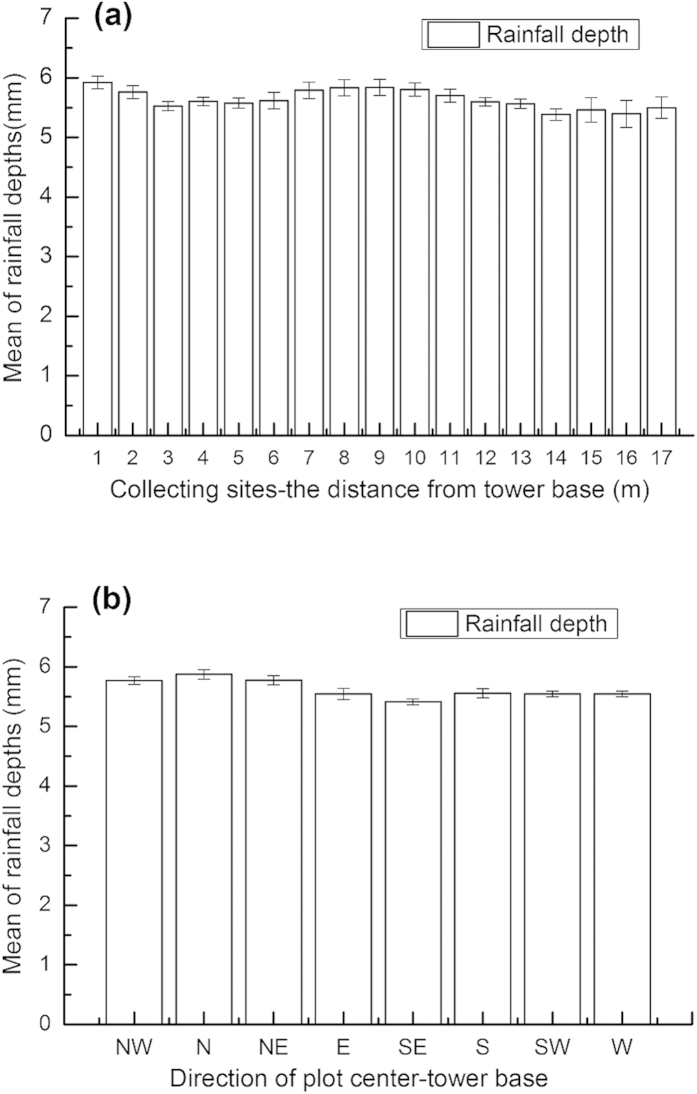
Preliminary testing result of the uniformity of the canopy spraying system. **(a)** The mean of spraying rainfall depths at a concentric circles (n = 8); **(b)** the mean of spraying rainfall depths at a 1 m interval along eight directions from the center to 17 m (n = 17). Error bars represent standard error of the means. NW, northwest; N, north; NE, northeast; E, east; SE, southeast; S, south; SW, southwest; W, west.

To test the uniformity of spraying solution, a temporary canopy spraying system as used in the CAN plots was implemented on an open ground. From the center of tower base around to eight direction, rainfall collectors were placed at a 1 m interval within 17 m (resulted in a total of 136 collectors). After 60 min experimental spraying, the mean of rainfall depths was 5.64 ± 0.03 mm (converted to rainfall capacity). There were no significant differences in spraying rainfall depths among the collecting sites (Fig. 3 a,b; *p* > 0.05). The preliminary testing results revealed that the canopy spraying system could ensure the uniformity of spraying solutions within a semi-diameter of 17 m under a calm weather condition (wind speed <1 m/s).

## Testable hypotheses and conceptual model

The approach-UAN is expected to result in a greater retention of added N within the understory and soil, comparing to approach-CAN with the same level of N treatment, which can in turn enlarge the effect size of simulated N deposition. Experiments with conventional UAN approaches may dramatically reduce understory species diversity resulted from exposure to toxic levels of N^14^. High concentration of N compounds could damage cell membrane, and limit stomatal conductance, which may decrease transpiration and photosynthesis of the understory leaves^52^. It has been reported that excessive N uptake can result in foliar necrosis, reduced drought and frost tolerance and increased susceptibility to pests and pathogens^53^. Excessive N uptake also has the potential to uncouple photophoshorylation, disrupt foliar acid/base regulation and create foliar cation deficiencies^27^. Such effects could also occur with CAN with concentrated treatment solutions. The approach-UAN may increase soil moisture more than the CAN treatment with same quantity of water for targetable solutions. Canopy interception redistributes water in space, creating patterns of distinct wet and dry spots which persist through time^54^. In addition, canopy-deposition interaction is the washing-off of dry deposited compounds (e.g., Na, Ca, Mg, and S) accumulated on the canopy^55^. Therefore, the conclusions drawn from the experiments with UAN should be extrapolated with caution.

**Figure 4:**
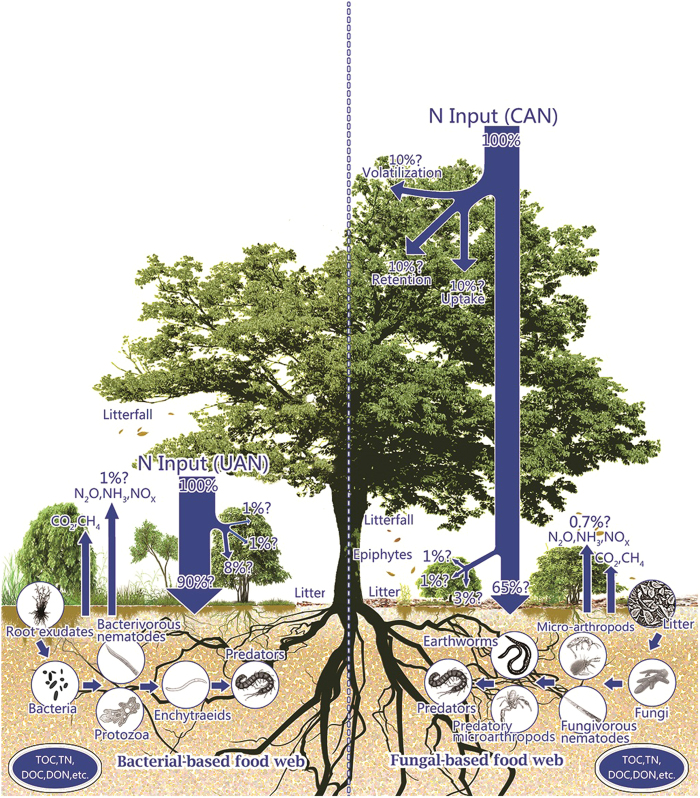
Conceptual model of responses of major structure and functions of forest ecosystem to CAN and UAN. The magnitude of the N fluxes, integrated over the whole forest ecosystem was adapted from the observations of canopy retention N investigation (referred to the text), suggesting that the canopies might retain the deposited N from the atmosphere. “?” indicated that the amount of redistributed N need further verification. Hypotheses on the responses of plants and soil food web to N solution via CAN or UAN approach please refer to the following text. The figure was created using Adobe Illustrator CS 5.0 and Adobe Photoshop CS 5.0 software. Figure drawing by X.Q. Rao.

We propose the following hypotheses: i) UAN might have overestimated the effects of N deposition on understory species and soil processes, but underestimated the effects on canopy-associated biota and processes, ii) the composition of inorganic N might be changed during the passage through forest canopy with NH_4_^+^ fraction being reduced whereas NO_3_^−^ being increased, iii) when low or moderate dose of N solution is used: CAN could favor the canopy tree species and other biota (e.g., epiphytes, lichens, mosses, algae, orchids etc.), resulting in greater leaf growth and more leaf litter. Greater litterfall mass could favor detritus food web, and thus fungal channel pathway dominates^56^,^57^,^58^. By contrast, UAN might favor the understory plant species and leads to greater root ingrowths and faster root turnover, which favors rhizosphere food web and bacterial channel pathway dominates^59^ (Fig. 4), and iv) when excess dose of N solution is used: CAN could damage the canopy tree species and other biota due to osmotic effects of salt, and consequently reducing growth of canopy tree species and other biota. However, the N in throughfall might be suitable for understory plant species and favors rhizosphere food web and bacterial channel pathway^56^,^57^,^59^. On the contrary, UAN could cause detrimental effects on understory plants and soil food web due to serious stress of high N input, resulting in reduced understory plant growth and damaged soil food web. Fungal channel pathway could dominate in response to greater litterfall inputs^58^ (Fig. 4).

Compared to UAN, CAN can be used to better illustrate the fate of the whole-forest ecosystem in response to elevated N deposition and to identify the major pathways and processes of N cycling (Fig. 4). CAN enables us to estimate the complete N budget by quantifying N inputs and outputs in forest ecosystems, where we need to explicitly include not only N added onto forest canopy but also N retained in the canopy, throughfall, stem-flow and leached out as soil solution. Most of these processes are neglected with UAN, which applies N fertilizer onto the understory and/or forest floors. To what extent the impacts of N deposition might have on the forest structure and functions, whether the canopy itself or the epiphytes it supports would produce similar response under sustained inputs over a long time. These issues could be fully addressed by CAN. The models for predicting forest ecosystems, which need to be further evaluated using actual field measurements and can only be improved with extensive data collection^60^, would be fine-tuned using the data collected via CAN. As feedback mechanisms and processes are considered at the whole ecosystem scale, CAN can provide the most suitable framework for prediction of forest ecosystems responding to elevated N deposition.

## Preliminary results of JGS forest site

To address the different responses of forest to CAN vs. UAN, multidisciplinary data is being collected including population ecology, ecophysiology and soil ecology. Data collection was initiated in 2013 or 2014 depending on parameters. The sampling methods and measurement procedures are presented in supplementary materials (Supplementary Info. S1). The preliminary data of JGS site are presented in diagrams. No detailed statistical analysis and in-depth discussions have been done since it is too early to make firm conclusion at this stage.

### The specific leaf area and leaf N contents

**Figure 5:**
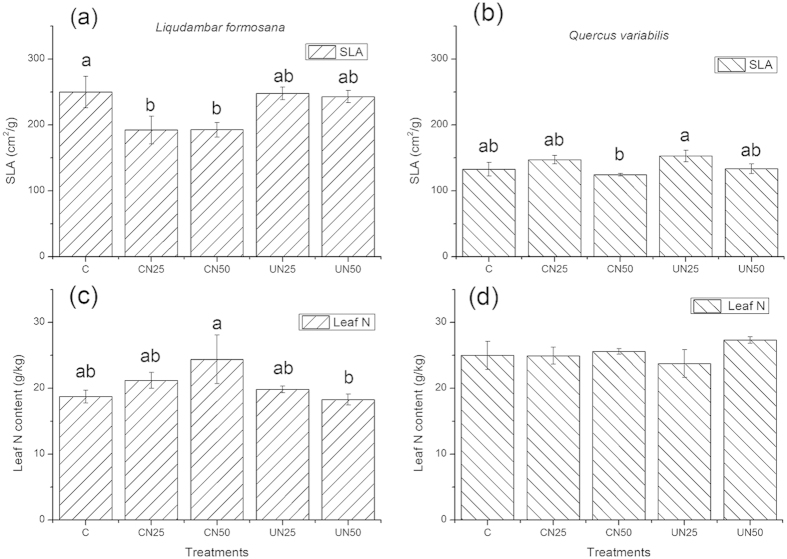
Specific leaf area (SLA) and leaf N contents for two canopy tree species in Jigongshan forest. Values are means ± SE. Different letters indicate significant differences at *p* < 0.05.

For a dominant canopy tree species *Liquidambar formosana*, CAN (CN25 and CN50) showed significant lower specific leaf area (SLA) compared to the control (C) and UAN plots (UN25 and UN50), among which no difference in SLA was found (Fig. 5a). Similarly, we found a significant lower SLA in CN50 for another canopy tree species *Quercus variabilis* (Fig. 5b). No differences in leaf N contents of the two species were found among treatments (Fig. 5c,d). In addition, there were no differences in SLA and leaf N contents among the five treatments for any understory tree species (data not shown). The results indicate that leaf morphological trait (i.e. SLA), rather than leaf N content, responds more quickly to N additions. SLA of canopy tree species decreased significantly under CAN (particularly CN50), but did not change under UAN treatments, indicating that conventional approach (i.e. UAN) might underestimate the impacts of N deposition on the canopy processes.

### Nitrous oxide emission and soil properties

**Figure 6:**
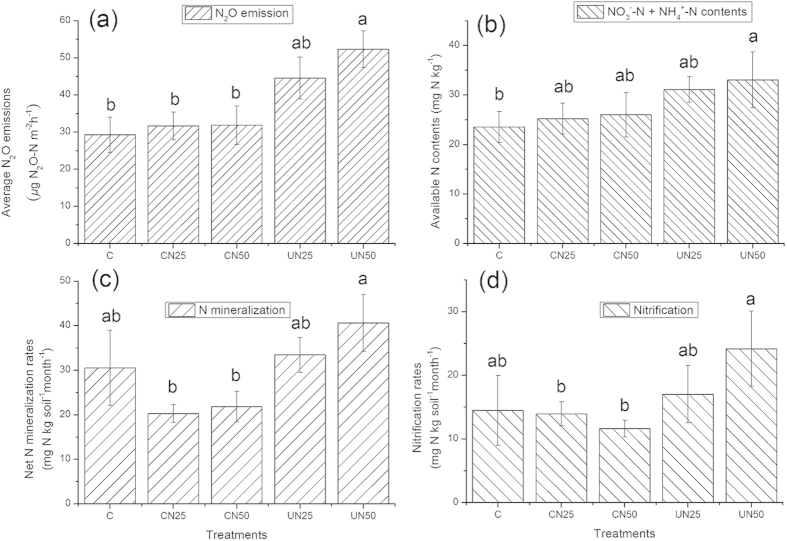
Average soil N_2_O emission rates (**a**), soil available N contents (**b**), net N-mineralization rate (**c**) and nitrification rate (**d**) of Jigongshan forest. Soil N_2_O fluxes were measured from April 2013 to November 2014. Values are means ± SE (n = 4). Different letters indicate significant differences at *p* < 0.05.

The average nitrous oxide (N_2_O) emissions significantly increased under UAN, but not change under CAN treatments relative to the controls (Fig. 6a, *p* = 0.04). UAN (especially in UN50) significantly increased soil available N (Fig. 6b, *p* = 0.03) whereas CAN had no effect. There were marginally significant increases in net N-mineralization and nitrification rates in the UAN plots (Fig. 6a,b, *p* = 0.07 and 0.06, respectively for net N-mineralization and nitrification). The increases in soil NH_4_^+^ and NO_3_^−^ contents, net N-mineralization and nitrification rates caused by adding N directly to the forest floor, might contribute to the increase in N_2_O emissions under the UAN treatments. The results indicate that canopy could lead to a lagging effect of N addition on soil N cycles.

### Abundance of ammonia-oxidizing archaeal and bacterial

**Figure 7:**
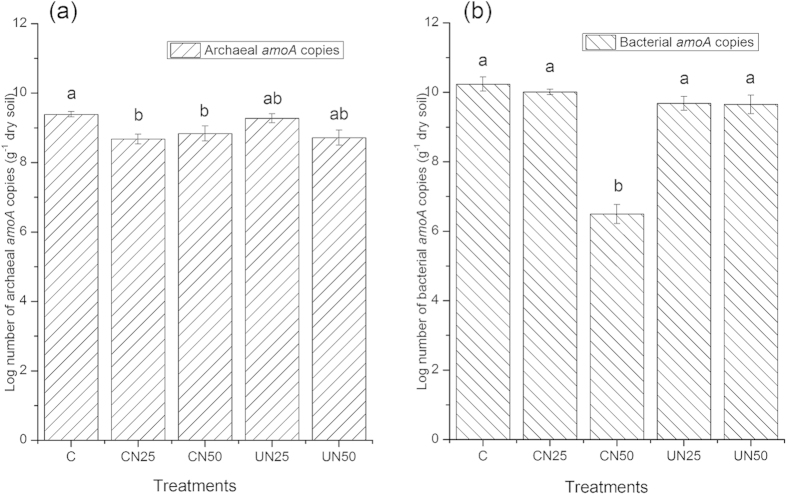
Abundance of archaeal (**a**) and bacterial (**b**) *amoA* gene copy numbers in Jigongshan forest. Values are means ± SE. Different letters indicate significant differences at *p* < 0.05.

Archeal *amoA* copies ranged from 2.0 × 10^8^ to 8.0 × 10^9^ across all treatments. Nitrogen addition significantly reduced ammonia-oxidizing (AOA) abundance in the UN50, CN25 and CN50 plots comparing to the control plots (Fig. 7a). Generally, ammonia-oxidizing bacterial (AOB) abundance was lower than AOA abundance, and ranged from 4.8 × 10^5^ to 1.3 × 10^8^. Nitrogen addition significantly deceased AOB abundance only in the CN50 plots (Fig. 7b). CAN treatments (especially in CN50) significantly reduced both AOA and AOB abundances. However, there was a marginally decrease in AOA abundance in the UAN plots. The results indicate that there were different responses of AOA and AOB abundances to the CAN vs. UAN treatments.

In conclusion, an experimental manipulation of CAN vs. UAN with a novel technology has been conducted at two latitudinal sites in China. The preliminary results provided many reliable evidences for the different responses of JGS forest to CAN vs. UAN treatments. The result indicated that the conventional approach-UAN might have neglected many important N cycling processes in forest canopy, making it difficult to accurately extrapolate the responses of forest ecosystem to atmospheric N deposition. The study, for the very first time to the best of our knowledge, designed a system to compare CAN with UAN and to examine the ecological impacts of N deposition on forest ecosystem from a holistic point view. The findings of this research program will improve not only the mechanistic understanding, but also model simulation and projection forest ecosystems in response to atmospheric N deposition in the future.

## Additional Information

**How to cite this article**: Zhang, W. *et al.* CAN Canopy Addition of Nitrogen Better Illustrate the Effect of Atmospheric Nitrogen Deposition on Forest Ecosystem? *Sci. Rep.* **5**, 11245; doi: 10.1038/srep11245 (2015).

## Supplementary Material

Supplementary Information

## Figures and Tables

**Table 1:** **Site and community structure characteristics of JGS and SMT forests.**

	**Site**	
**Characteristic**	**JGS**	**SMT**
Forest type	Mixed deciduous forest	Broadleaved evergreen forest
Annual rainfall (mm)	1119	2364
Annual temperature(^o^C)	15.2	20.8
N deposition in rainfall	19.6 (kg N ha^−1^yr^−1^)	34.1 (kg N ha^−1^yr^−1^)
Soil type	Yellow-brown soil	Latosolic red soil
Forest age (year)	45 (thinned in 1970)	50 (thinned in 1965)
Stand density (tree ha^−1^)	446	818
Mean DBH (cm)	26.6	18.6
Mean tree height (m)	21.6	13.8
Dominant species	*Quercus acutissima* Carruth.; *Quercus variabilis* Bl.; *Liquidambar formosana* Hance.	*Cryptocarya concinna*; *Schima superba*; *Machilus chinensis*; *Castanea henryi (Skan)* Rehd.; *Engelhardtia roxburghiana*

Data of temperature and rainfall were collected from nearby meteorological stations, and the values are mean of last 60 years. N deposition in rainfall was minored from April 2011 to March 2013 at both forest sites. The trees with DBH ≥ 10 cm were investigated on July 2012. DBH, diameter at breast height; JGS, Jigongshan Forest; SMT, Shimentai Forest.
