# Supplementary material for: CAN Canopy Addition of Nitrogen Better Illustrate the Effect of Atmospheric Nitrogen Deposition on Forest Ecosystem?
Source: Sci Rep. 2015 Jun 10;5:11245. doi: 10.1038/srep11245 (PMC4462050; doi:10.1038/srep11245)
Supplement: Supplementary Information [file srep11245-s1.pdf]

SUPPLEMENTARY INFORMATION

Title: CAN Canopy Addition of Nitrogen Better Illustrate the Effect of Atmospheric Nitrogen Deposition on Forest Ecosystem?

Running title: Canopy Nitrogen Deposition on Forests

Wei Zhang[1,3§], Weijun Shen[1§], Shidan Zhu[1], Shiqiang Wan[2], Yiqi Luo[3], Junhua Yan[1], Keya Wang[1], Lei Liu[1], Huitang Dai[4], Peixue Li[4], Keyuan Dai[5], Weixin Zhang[1], Zhanfeng Liu[1], Faming Wang[1], Yuanwen Kuang[1], Zhian Li[1], Yongbiao Lin[1], Xingquan Rao[1], Jiong Li[1], Bi Zou[1], Xian Cai[1], Jiangming Mo[1], Ping Zhao[1], Qing Ye[1], Jianguo Huang[1] & Shenglei Fu[1*]

[1]Key Laboratory of Vegetation Restoration and Management of Degraded Ecosystems, South China Botanical Garden, Chinese Academy of Sciences, Guangzhou 510650, China

[2]State Key Laboratory of Cotton Biology, Key Laboratory of Plant Stress Biology, College of Life Sciences, Henan University, Kaifeng, Henan 475004, China

[3]Department of Microbiology and Plant Biology, University of Oklahoma, Norman, OK 73019, USA

[4]Jigongshan National Natural Reserve, Xinyang, Henan 464000, China

[5]Shimentai National Natural Reserve, Yingde 513000, China

[§]These authors contributed equally to this work.

***Corresponding author:** Dr. Shenglei Fu

South China Botanical Garden, Chinese Academy of Sciences

Address: 723 Xingke Road, Tianhe District, Guangzhou 51065, China

Email: sfu@scbg.ac.cn; Phone: +86-20-37252722; Fax: +86-20-37252711

Number of tables: 1

Number of figures: 7

Number of reference: 60

Supplementary information:

Sampling methods and measurement procedures

The specific leaf area and leaf N contents

Two dominant canopy tree species (*Liquidambar formosana* and *Quercus variabilis*) were selected for measurements, and five individuals were sampled per species for each treatment. In November 2014 (after two years treatment), 30-50 healthy and sunny leaves were sampled from each individual. Leaf area was measured with a leaf area meter (Li-3000A, Li-Cor, Nebraska, USA), then the leaves were oven-dried for 48 h at 70 ℃ to determine the dry mass. Specific leaf area (SLA) was calculated as leaf area per dry mass. The dry leaves were ground and homogenized for chemical analyses. Mass-based total N content was determined by Kjeldhal analysis.

Nitrous oxide emission and soil properties

Soil nitrous oxide (N_2O) emissions were measured since April 2013 using a static chamber method. Gas samples were collected biweekly during growth season (April to September) and monthly at other times. The chamber design and the measurement procedure were adopted from Zhang et al. (2014)[1]. Soil samples were collected in July 2014 for analyzing properties. Five soil cores (3.5 cm diameter) were collected randomly from each plot at 0-10 cm depth and combined to one composite sample for each plot. Soil available N (NH_4^+ and NO_3^-) contents were determined by extraction with 2 M KCl solution followed by colorimetric analysis on a flow-injection autoanalyzer (Lachat Instruments, Milwaukee, USA). From 1 to 30 July 2014, the rates of soil net N-mineralization and nitrification were measured using an intact core incubation. Six soil cores (0-5 cm depth, 3.5 cm diameter) were sampled from each plot. Three cores were brought to the lab for extraction of available N contents, and the others were returned to the plot for *in situ* incubation[2]. Nitrification rate was calculated from the difference between extractable NO_3^- contents before and after incubation, and net N mineralization rate was calculated as the accumulation of total inorganic N over the incubation[2]. The data were expressed as mg N kg^{-1} dry weight soil $month^{-1}$.

Abundance of ammonia-oxidizing archaeal and bacterial

Soil samples were collected in December 2013 (after 8 months treatment). From each plot, five soil cores (5 cm in diameter) were collected randomly from 0-10 cm depth and combined to one composite sample.

Soil DNA was extracted from 0.25 g of soil using the Power-Soil DNA Isolation Kit (MoBio Laboratories Inc., Carlsbad, CA, USA). The concentration and purity of the extracted DNA was assessed using a Nanodrop® ND-2000 UV-Vis Spectrophotometer (NanoDrop Technologies, Wilmington, DE). The abundances of ammonia-oxidizing archaeal (AOA) and ammonia-oxidizing bacterial (AOB) were estimated by quantifying the *amoA* genes, which determined by qPCR using the primer pairs Arch-amoAF/Arch-amoAR[3] and amoA1F/amoA2R[4], respectively.

References:

1 Zhang, W. *et al.* Responses of nitrous oxide emissions to nitrogen and phosphorus additions in two tropical plantations with N-fixing vs. non-N-fixing tree species. *Biogeosciences* **11**, 4941-4951, doi:10.5194/bg-11-4941-2014 (2014).

2 Zhu, W. X. & Carreiro, M. M. Chemoautotrophic nitrification in acidic forest soils along an urban-to-rural transect. *Soil Biol Biochem* **31**, 1091-1100, doi:10.1016/S0038-0717(99)00025-5 (1999).

3 Francis, C. A., Roberts, K. J., Beman, J. M., Santoro, A. E. & Oakley, B. B. Ubiquity and diversity of ammonia-oxidizing archaea in water columns and sediments of the ocean. *P Natl Acad Sci USA* **102**, 14683-14688, doi:10.1073/pnas.0506625102 (2005).

4 Rotthauwe, J. H., Witzel, K. P. & Liesack, W. The ammonia monooxygenase structural gene amoA as a functional marker: Molecular fine-scale analysis of natural ammonia-oxidizing populations. *Appl Environ Microb* **63**, 4704-4712 (1997).
